# Neurocognitive risk markers in first-episode major depressive disorder with positive family history: a large-scale case–control study

**DOI:** 10.3389/fpsyt.2025.1662007

**Published:** 2026-01-14

**Authors:** Zhiyong Li, Min Pan, Xulai Zhang, Anzhen Wang, Wenmei Fang, Jianjun Guan, Boyu Zhang, Xialong Cheng

**Affiliations:** 1 Department of Psychiatry, The Affiliated Psychological Hospital of Anhui Medical University, Hefei, China; 2 Department of Psychiatry, Hefei Fourth People’s Hospital, Hefei, China; 3 Department of Clinical Psychology, Anhui Mental Health Center, Hefei, China; 4 Department of Mental Health Research, Anhui Clinical Research Center for Mental Disorders, Hefei, China

**Keywords:** major depressive disorder, family history, RBANS, neurocognition, risk marker

## Abstract

**Objective:**

To identify specific neurocognitive risk markers in first-episode major depressive disorder (MDD) patients with positive family history (PFH).

**Methods:**

Antipsychotic-naive adults aged 18–60 were recruited across three groups: major depressive disorder patients with positive family history (PFH-MDD, *n* = 171), major depressive disorder patients with negative family history (NFH-MDD, *n* = 185), and healthy controls (HCs, *n* = 180). All patients met the DSM-5 criteria for first-episode MDD (HAMD-24 ≥ 17). Neurocognition was assessed with the Repeatable Battery for the Assessment of Neuropsychological Status (RBANS). Group differences were examined using the Kruskal–Wallis test and ANCOVA. Logistic regressions identified independent cognitive predictors; ROC curves evaluated discriminative validity.

**Results:**

The RBANS total and domain scores differed across the groups (*p* < 0.001). PFH-MDD performed worse than NFH-MDD in language function (*p* < 0.001) and total score (*p* < 0.001). In the PFH group, language function score was negatively correlated with HAMD score (*r* = −0.184, *p* = 0.016). In the NFH group, language function score was positively correlated with HAMA score (*r* = 0.402, *p* < 0.001) and negatively correlated with HAMD score (*r* = −0.364, *p* < 0.001). Total score was negatively correlated with HAMD score (*r* = −0.158, *p* = 0.032). After adjustment, language function (OR = 0.82, *p* = 0.042) and total score (OR = 0.90, *p* < 0.001) independently predicted PFH-MDD; only total score predicted NFH-MDD (OR = 0.77, *p* < 0.001). The ROC-AUC values for PFH-MDD were as follows: language = 0.967 and total score = 0.991. Gender × group interactions were non-significant.

**Conclusions:**

Language dysfunction and global cognitive impairment may be independent markers of first-episode MDD with PFH. Early cognitive profiling may facilitate targeted prevention in high-risk relatives.

## Introduction

Major depressive disorder is highly heritable (*h*² ≈ 40%–70%) (1). Meta-analyses of never-depressed first-degree relatives demonstrate small to medium deficits in intelligence, memory, and language (2, 3). Whether these deficits represent premorbid vulnerability markers or epiphenomena remains unresolved (4). We hypothesized that antipsychotic-naive first-episode major depressive disorder (MDD) patients with positive family history (PFH) would display a distinct cognitive signature predictive of disorder onset (5).

## Methods

### Participants

A large-scale case–control study was conducted in August 2020 and June 2023 at Hefei Fourth People’s Hospital. The inclusion criteria were as follows: 1) age 18–60 years, 2) DSM-5 first-episode MDD (SCID-5), 3) HAMD-24 ≥17, 4) PFH defined as ≥1 first-degree relative with DSM-5 MDD confirmed by hospital records, 5) no psychotropic medication, and 6) signed informed consent. The exclusion criteria include recurrent depression, other axis I disorders, ADHD, neurological illness, and substance dependence. Healthy controls (HCs) were community volunteers matched for age, gender, parental education, and estimated IQ (WASI-2). This study was approved by the Ethics Committee of the Fourth People’s Hospital of Hefei (No. HFSY-IRB-YJ-KYXM-CL.2024-064-001).

### Clinical assessments

HAMD-24 (cutoff ≥ 17) and HAMA (cutoff ≥ 14) were validated in China. RBANS includes five index scores + total score (normative Chinese version; higher scores = better performance). Interrater ICC was >0.90.

### Statistical analysis

SPSS 22.0 software was used for the statistical analysis. PASS 11.0 software was used to calculate the sample size. The measurement data conforming to the normal distribution are represented as mean ± SD. Count data conforming to a non-normal distribution were expressed as [*M* (P25, P75)]. Power analysis (GPower 3.1) indicated *n* = 171 per group to detect *d* = 0.30 at 90% power. Demographics were compared using one-way ANOVA or *χ*²; *post hoc* comparisons employed Tukey’s test. For RBANS, group differences were assessed using the Kruskal–Wallis test with Bonferroni *post hoc* corrections when appropriate. ANCOVA was used to compare groups controlling for gender, parental education, and IQ. Associations between variables were evaluated by Spearman’s *ρ* with FDR correction (*q* < 0.05). The Spearman’s correlation test was used to examine the correlation between the neurocognitive function in five dimensions (immediate memory, visual span, language function, attention, and delayed memory) with psychiatric symptoms (HAMA and HAMD scores). Logistic regression (enter) was adjusted for the above covariates; the Hosmer–Lemeshow value was >0.05, indicating a good fit. ROC analysis was used to establish optimal cut-points based on the Youden index. Significance was defined as two-tailed *p* < 0.05 (FDR-corrected for 20 primary tests).

## Results

### Sample characteristics

There were no significant differences in age, gender, years of education, or symptom severity among the groups (**Table 1**).

**Table 1:** Social demographic data of PFH-MDD, NFH-MDD, and HCs (mean ± SD).

Group	n	Age (years)	Gender (*n*)		Years of education (years)	Average duration (weeks)	HAMA (points)	HAMD (points)
			Male	Female				
PFH-MDD	171	31.8 ± 7.9	56	115	12.4 ± 1.7	18.5 ± 12.1	18.24 ± 5.13	26.5 ± 4.7
NFH-MDD	185	32.8 ± 8.6	60	125	12.7 ± 2.5	16.4 ± 11.7	16.94 ± 4.73	28.4 ± 4.4
HCs	180	34.6 ± 9.5	62	118	13.5 ± 3.3			

### RBANS performance

PFH-MDD < NFH-MDD < HCs across all domains (*p* < 0.001). NFH was significantly higher than PFH on language function score and total score (*p* < 0.001). Effect sizes remained significant after covariate adjustment. Gender × group interactions were non-significant for all domains (*p* > 0.05) (**Tables 2**, **3**).

**Table 2:** Comparison of the RBANS test among the three groups [*M* (P25, P75), points].

Neuropsychological status battery (RBANS)	PFH-MDD	NFH-MDD	HCs	*p*-value
	(*n* = 171)	(*n* = 185)	(*n* = 180)	
Immediate memory	35 (19, 45)	34 (28, 40)	52 (48, 56)	<0.001^**^
Visual span	36 (33, 37)	35 (34, 37)	44 (39, 55)	<0.001^**^
Language function	25 (17, 28)	32 (29, 34)	39 (36, 44)	<0.001^**^
Attention	48 (18, 58)	51 (48, 53)	73 (67, 77)	<0.001^**^
Delayed memory	42 (40, 48)	39 (32, 53)	57 (54, 60)	<0.001^**^
Total score	174 (157, 199)	192 (179, 201)	276 (261, 283)	<0.001^**^

^*^p < 0.05, ^**^p < 0.001.

**Table 3:** Comparison of RBANS test scores between two groups.

Neuropsychological status battery (RBANS)	PFH-MDD vs. NFH-MDD		PFH-MDD vs. HCs		NFH-MDD vs. HCs	
	*Z*	*p*	*Z*	*p*	*Z*	*p*
Immediate memory	1.084	0.835	−13.883	<0.001^**^	−15.259	<0.001^**^
Visual span	1.375	0.508	−13.749	<0.001^**^	−15.417	<0.001^**^
Language function	−8.233	<0.001^**^	−10.292	<0.001^**^	−18.269	<0.001^**^
Attention	−1.349	0.177	−14.375	<0.001^**^	−15.434	<0.001^**^
Delayed memory	1.172	0.723	−12.535	<0.001^**^	−13.973	<0.001^**^
Total score	−3.805	<0.001^**^	−14.574	<0.001^**^	−18.068	<0.001^**^

^*^p < 0.05, ^**^p < 0.001.

### Correlation analysis between RBANS scores (language function, total score) and HAMA and HAMD scores in PFH-MDD and NFH-MDD

In the PFH group, language function score was negatively correlated with HAMD score (*r* = −0.184, *p* = 0.016). In the NFH group, language function score was positively correlated with HAMA score (*r* = 0.402, *p* < 0.001) and negatively correlated with HAMD score (*r* = −0.364, *p* < 0.001). Total score was negatively correlated with HAMD score (*r* = −0.158, *p* = 0.032) (**Table 4**).

**Table 4:** Correlation analysis between RBANS scores (language function, total score) and HAMA and HAMD scores in PFH-MDD and NFH-MDD.

Scale scores	PFH-MDD				NFH-MDD			
	Language function		Total score		Language function		Total score	
	*r*	*p*	*r*	*p*	*r*	*p*	*r*	*p*
HAMA	0.107	0.164	−0.04	0.607	0.402	<0.001^**^	−0.143	0.052
HAMD	−0.184	0.016^*^	−0.027	0.728	−0.364	<0.001^**^	−0.158	0.032^*^

^*^p < 0.05, ^**^p < 0.001.

### Cognitive predictors of group membership

Language function and total score independently predicted PFH-MDD *vs*. HCs (OR = −0.82 and −0.90); only total score predicted NFH-MDD *vs*. HCs (OR = −0.77). The results were corrected by FDR (**Table 5**).

**Table 5:** Binary logistic regression analysis of neurocognitive risk marks of major depressive patients.

RBANS scores	PFH-MDD and HCs				NFH-MDD and HCs			
	OR	95% CI		*p*	OR	95% CI		*P*
		*Z* (0.025)	*Z* (0.975)			*Z* (0.025)	*Z* (0.975)	
Language function	−0.82	0.676	0.993	0.042^*^	−0.93	0.716	1.214	0.604
Total score	−0.90	0.853	0.942	<0.001^**^	−0.77	0.675	0.867	<0.001^**^

^*^p < 0.05, ^**^p < 0.001.

### ROC analysis

**Figure 1:**
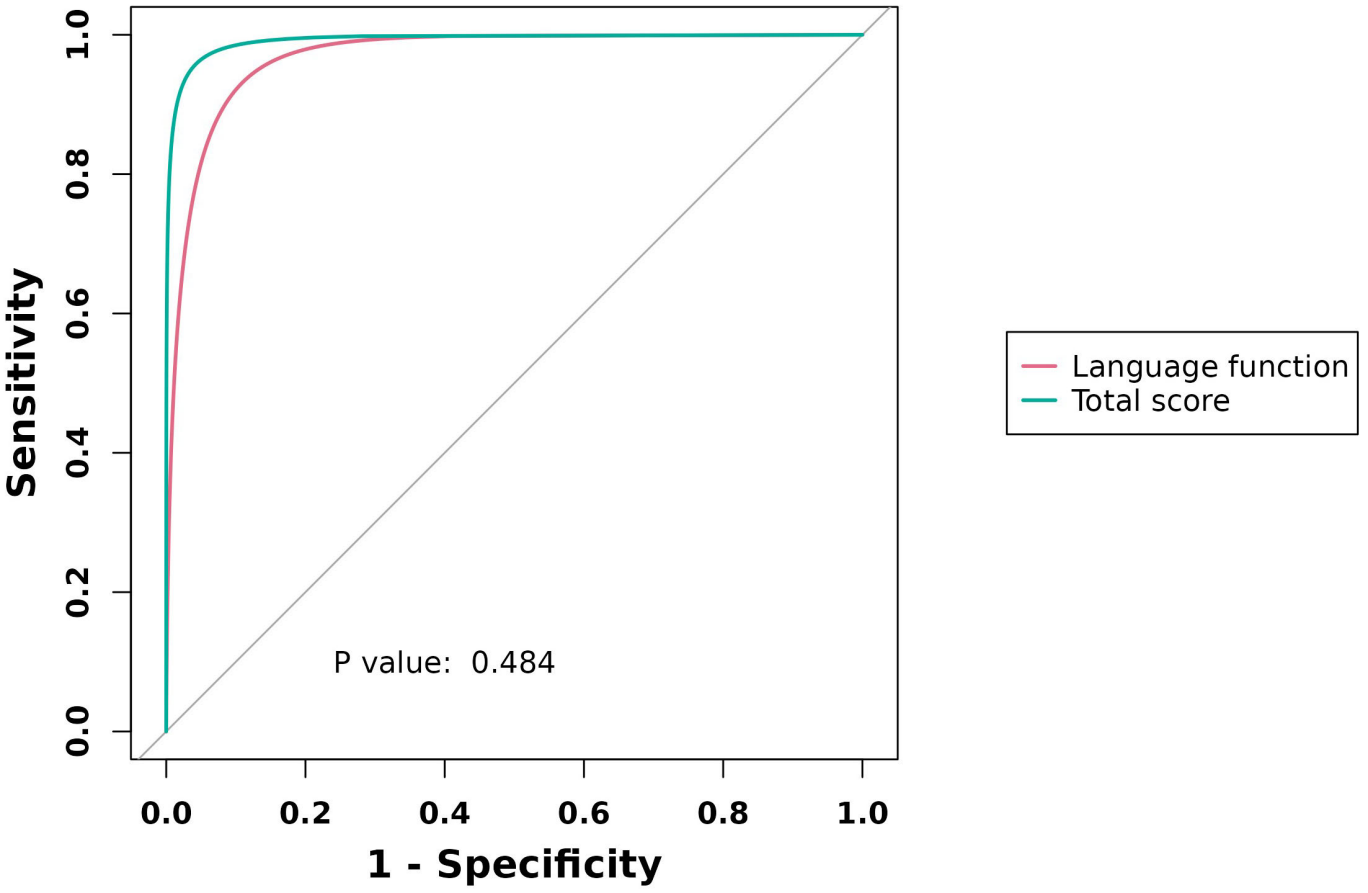
ROC curve analysis of RBANS (language function, total score) in PFHG vs. HCs.

**Figure 2:**
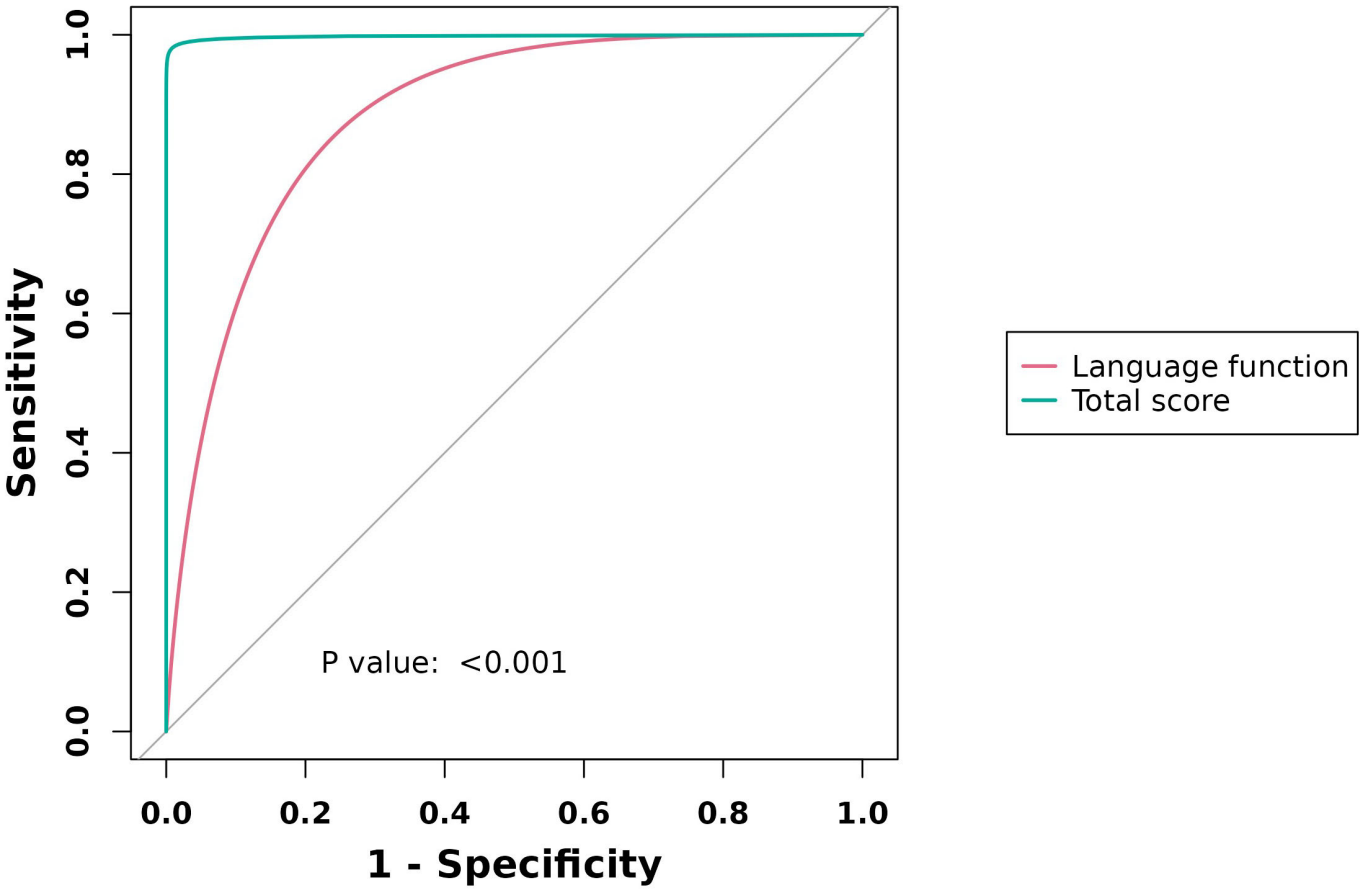
ROC curve analysis of RBANS (language function, total score) in NFHG vs. HCs.

The AUC values for PFH-MDD were as follows: language = 0.967 (95% CI: 0.837–0.982) and total score = 0.991 (95% CI: 0.967–0.997). For NFH-MDD, the AUC values were as follows: language = 0.883 (95% CI: 0.846–0.917) and total score = 0.997 (95% CI: 0.983–0.998) (**Table 6**; **Figures 1**, **2**).

**Table 6:** ROC curve analysis of RBANS (language function, total score) in major depressive patients.

Group	Neuropsychological status battery (RBANS)	AUC (95% CI)	Sensitivity (%)	Specificity (%)	Cut-point
PFH-MDD	Language function	0.967 (0.837, 0.982)	98.80	87.80	34
	Total score	0.991 (0.967, 0.997)	96.50	98.90	224
NFH-MDD	Language function	0.883 (0.846, 0.917)	77.80	87.80	34
	Total score	0.997 (0.983, 0.998)	99.50	98.90	229

## Discussion

We examined neurocognitive profiles in 536 antipsychotic-naive adults experiencing their first major depressive episode. Patients with a positive family history of MDD (PFH-MDD, *n* = 171) showed significantly lower language and global RBANS scores than family-history-negative patients (NFH-MDD, *n* = 185) and HCs (*n* = 180) (6). Language and total scores survived adjustment for gender, parental education, and estimated IQ; predicted PFH-MDD *vs*. HCs with excellent discrimination (AUC ≥ 0.96); and were selectively correlated with symptom severity in PFH-MDD (7). These data indicate that language dysfunction is a robust, independent marker of familial risk for depression and may represent a target for early identification and preventive intervention (8).

The heritability of MDD is 40%–70% (1). By restricting the sample to first-episode, medication-free patients, we removed confounds of illness chronicity and treatment, allowing purer estimation of genetic load (9). The effect size for language impairment in PFH-MDD (Cohen’s *d* = 0.48) was more than twice that in NFH-MDD (*d* = 0.22), supporting a quantitative gene–cognition pathway rather than a simple “exposed *vs*. non-exposed” dichotomy (10). This gradient is consistent with recent polygenic-risk studies demonstrating that greater MDD polygenic scores are associated with reduced verbal fluency in the general population (11). Language tasks simultaneously recruit left inferior frontal gyrus, temporal pole, and inferior parietal lobule—regions showing hypoactivation during verbal fluency in drug-naive MDD (12), reduced cortical thickness in high-risk offspring (13), and oligodendrocyte-related gene downregulation in postmortem MDD (14). Thus, language dysfunction may mirror early neurodevelopmental alterations driven by oligodendroglial–synaptic genes implicated in MDD heritability (15).

From a practical standpoint, the RBANS language subtest requires <5 min, can be administered on paper or digitally, and is culture-fair in Chinese populations (16).A cutoff ≤28 yielded 96% sensitivity and 88% specificity for PFH-MDD in our ROC analysis. Embedding this brief screen in university or primary care mental health checkups could flag high-risk young adults before syndromal onset (17). Secondly, language-based cognitive training (e.g., semantic category generation, phonemic switching) has improved executive functions and functional outcome in established MDD (18); our findings justify testing such interventions in the prodromal phase (19). Negative studies often included recurrent cases, used coarse instruments (MMSE), or failed to control for IQ and parental education (20). We minimized these biases by recruiting only first-episode, medication-free participants, adjusting for estimated IQ and parental education, and correcting for 20 primary cognitive comparisons with FDR (21). The absence of gender × group interactions further suggests that our results are generalizable across sexes (22).

## Limitations and future directions

The cross-sectional design precludes causal inferences; a 24-month follow-up of the present cohort is underway to determine whether language deficits predict conversion to MDD in high-risk relatives (23). RBANS is a screening battery; future work should incorporate comprehensive executive function and social cognition tasks (e.g., D-KEFS, hinting task) and digital phenotyping (24). Polygenic risk scores and epigenetic markers will be integrated to dissect gene–environment interactions underlying cognitive vulnerability (25). Replication in multi-ethnic samples is needed to confirm culture generalizability (26).

## Data Availability

The raw data supporting the conclusions of this article will be made available by the authors, without undue reservation.
